# Overexpression of Wheat Selenium-Binding Protein Gene *TaSBP-A* Enhances Plant Growth and Grain Selenium Accumulation under Spraying Sodium Selenite

**DOI:** 10.3390/ijms25137007

**Published:** 2024-06-26

**Authors:** Tongtong Xiao, Jian Qiang, Haocheng Sun, Fei Luo, Xiaohui Li, Yueming Yan

**Affiliations:** College of Life Science, Capital Normal University, Beijing 100048, China

**Keywords:** wheat, selenium-binding protein, selenium enrichment, molecular docking, transcriptome, biofor-tification

## Abstract

Selenium (Se) is an essential trace element for humans. Low concentrations of Se can promote plant growth and development. Enhancing grain yield and crop Se content is significant, as major food crops generally have low Se content. Studies have shown that Se biofortification can significantly increase Se content in plant tissues. In this study, the genetic transformation of wheat was conducted to evaluate the agronomic traits of non-transgenic control and transgenic wheat before and after Se application. Se content, speciation, and transfer coefficients in wheat grains were detected. Molecular docking simulations and transcriptome data were utilized to explore the effects of selenium-binding protein-A *TaSBP-A* on wheat growth and grain Se accumulation and transport. The results showed that *TaSBP-A* gene overexpression significantly increased plant height (by 18.50%), number of spikelets (by 11.74%), and number of grains in a spike (by 35.66%) in wheat. Under normal growth conditions, Se content in transgenic wheat grains did not change significantly, but after applying sodium selenite, Se content in transgenic wheat grains significantly increased. Analysis of Se speciation revealed that organic forms of selenomethionine (SeMet) and selenocysteine (SeCys) predominated in both W48 and transgenic wheat grains. Moreover, *TaSBP-A* significantly increased the transfer coefficients of Se from solution to roots and from flag leaves to grains. Additionally, it was found that with the increase in *TaSBP-A* gene overexpression levels in transgenic wheat, the transfer coefficient of Se from flag leaves to grains also increased.

## 1. Introduction

Wheat, together with maize and rice, is among the “three principal food crops globally” and stands as one of the first crops to be domesticated by humans, exhibiting robust adaptability and substantial yield potential [1]. As the primary nourishment for approximately 35% of the global population and a vital protein source, wheat flour can be processed into a variety of specialized foodstuffs such as bread, noodles, biscuits, and cakes, accommodating the diverse dietary preferences across different regions. Wheat grains contain abundant starches and proteins, as well as dietary fiber, vitamins, trace minerals, and advantageous phytochemicals, serving as an essential energy source [2].

Selenium is a non-metallic element categorized under leading group VI (element 34). Selenium naturally manifests in two distinct forms, organic and inorganic [3]. In its organic form, selenium predominantly occurs as seleno-amino acids or selenoproteins; meanwhile, its inorganic state includes the divalent, tetravalent, and hexavalent solids in soils and minerals, or the free chloride ions within organisms, encompassing a range of archaea, bacteria, protozoa, algae, certain plants, and nearly all animal species. The World Health Organization and the International Organization for Nutrition have identified selenium as an indispensable trace element for both humans and animals, advocating for an adult daily intake ranging from 50 to 200 μg [4]. In humans, selenium intake primarily occurs through the consumption of plant-based foods. Owing to its critical role as a trace element, selenium makes a significant contribution to human health, offering antioxidative, anticancer, and immunostimulatory benefits [5,6]. Additionally, selenium is also metabolized into selenocysteine, which impedes protein synthesis, thus obstructing the proliferation of cancer cells and inducing apoptosis [7]. Moreover, selenium enhances immunity by bolstering the bactericidal capacity of macrophages [8]. In regions with selenium-deficient soils, the scarcity of selenium can contribute to an array of health issues, including Creutzfeldt–Jakob disease, myocardial infarction, Alzheimer’s disease, and chronic pancreatitis [9]. 

Recent studies have shown that low concentrations of selenium have positive effects on plant growth, development, and yield. Shekari et al. found that selenium can promote root growth and increase leaf area in peppers, ultimately enhancing plant growth and biomass [10]. Chauhan et al. discovered that low doses of selenium can stimulate rice growth [11]. Additionally, the application of selenium as a fertilizer has been shown to promote crop growth and development. Selenium also acts as both an antioxidant and a pro-oxidant in plants, enhancing the synthesis of photosynthetic pigments, increasing the photosynthetic rate, gas exchange rate, accumulation of osmoprotectants, and production of various secondary metabolites. These functions endow plants with tolerance to various abiotic stresses, including salinity, drought, extreme temperatures, and toxic metal/metalloid stress [12,13,14,15]. In plants, selenium exists in the following three forms—organic, inorganic, and volatile—with inorganic selenium predominantly existing in the form of selenate and selenite. Plants are capable of absorbing selenium in the forms of selenate, selenite, and organic selenium [16]. Selenite is primarily absorbed by plants through the silicon endocytosis transporter and potentially via the phosphate transporter through the mechanism of metabolism-dependent active processes, leading to its accumulation in the root system with minimal translocation to the aerial parts. Conversely, selenate is absorbed by root cells through the high-affinity sulfate transporter located on the plasma membrane and is efficiently transported from the roots to the shoots through the xylem [17].

SBPs are a widely present and highly conserved protein family, belonging to the SBP56 family [18]. They are cytoplasmic proteins believed to be involved in the later stages of protein transport within the Golgi apparatus [19]. In mammals, *SBP1* has been found to be intricately linked to the development of cancer [20,21]. In the model plant *Arabidopsis*, the *SBP* gene family is comprised of three genes (*AtSBP1*, *AtSBP2*, and *AtSBP3*), among which *AtSBP1* is the most prominently expressed [22]. Previously, a specific Se-binding site, Cys21Cys22, was identified in AtSBP1, which is capable of reducing SeO_3_^2−^ to form [R-S-Se(II)-S-R] analogs [23]. In addition, SBPs have a putative heavy metal-binding motif, CXXC, which is highly conserved among different plant species. Proteins containing this motif are often involved in the metabolism and detoxification of metal ions [24,25]. Many studies have shown that *AtSBP1* responds to a variety of stressors in *Arabidopsis* seedlings, including Cd^2+^, Cu^2+^, Zn^2+^, selenate, selenite, and H_2_O_2_ [23]. Comparative studies involving the wheat genome identified three *TaSBP* gene sequences that are highly similar to *AtSBP1*, with *TaSBP-A* being particularly involved in Cd tolerance [26]. 

Currently, the primary method of artificially increasing plant selenium content is via selenium agronomic biofortification. The three common means of biofortification via foliar sprays, soil fertilization, and seed dipping. Selenite and selenate represent the predominant forms of selenium supplementation [27]. The comparative analyses between foliar application and soil irrigation with identical concentrations of sodium selenate and sodium selenite have demonstrated that foliar spraying significantly outperforms soil irrigation in both selenium fertilizer utilization and the ultimate efficiency of selenium enrichment in seeds. This discrepancy primarily arises due to the influence of the soil’s pH and the presence of other elements, such as iron, sulfur, and nitrogen, which can interfere with selenium absorption in the root system [17]. A study of two purple-grain wheat varieties examining foliar applications of sodium selenite at varying concentrations of selenium fertilizer determined that a dosage of 37.5 g/ha of sodium selenite facilitated the highest efficiency of selenium utilization [28]. Wang et al. conducted an analysis of selenium content in grains by applying different doses and valence states of foliar selenium fertilizer at various growth stages. They recommended applying 20 g/ha of sodium selenate before grain filling, which can increase the selenium content in grains to 134 μg/kg [29].

Wheat is an excellent selenium-enriched material. However, 63% of the wheat in China is selenium-deficient, and the average selenium concentration in wheat kernels is 64.6 μg/kg, which cannot meet the population’s health demand for selenium [30]. The most effective method for increasing grain selenium accumulation is to develop new wheat cultivars with a high capacity for selenium enrichment through biotechnological means. At present, the roles and molecular mechanisms of SBP proteins involved in the selenium enrichment of wheat grain remain unclear. In this research, we aimed to investigate the efficiency of selenium absorption in wheat grains through the overexpression of the *TaSBP-A* gene and foliar spraying using a sodium selenite solution. The study aimed to uncover the role of the *TaSBP-A* gene in selenium enrichment within wheat grains, contributing to the biofortification of crop selenium.

## 2. Results

### 2.1. Identification of TaSBP-A Transgenic Wheat

Three genetically stable wheat lines overexpressing the *TaSBP-A* gene were obtained through the genetic transformation of wheat variety W48, consecutive screening and identification, and then designated as OE-1, OE-2, and OE-3. Genomic polymerase chain reaction (PCR) analysis was conducted on the foliage DNA of seven randomly selected plants from each overexpression line, with W48 serving as the negative control. The results confirmed the presence of the *TaSBP-A* transgene in all three overexpressing lines (Figure S1A). Additionally, the resistance of seven randomly selected mature seeds from both W48 and the three overexpression lines was evaluated using PAT/bar speed test papers. The results indicated that, in W48, only the C-line bands were observed, whereas both the C- and T-line bands were detected in the overexpressing lines (Figure S1B). Consequently, these findings substantiated that the three lines belonged to genetically stable transgenic wheats.

### 2.2. Expression Analysis of TaSBP-A Gene in Different Organs of Wheat

**Figure 1:**
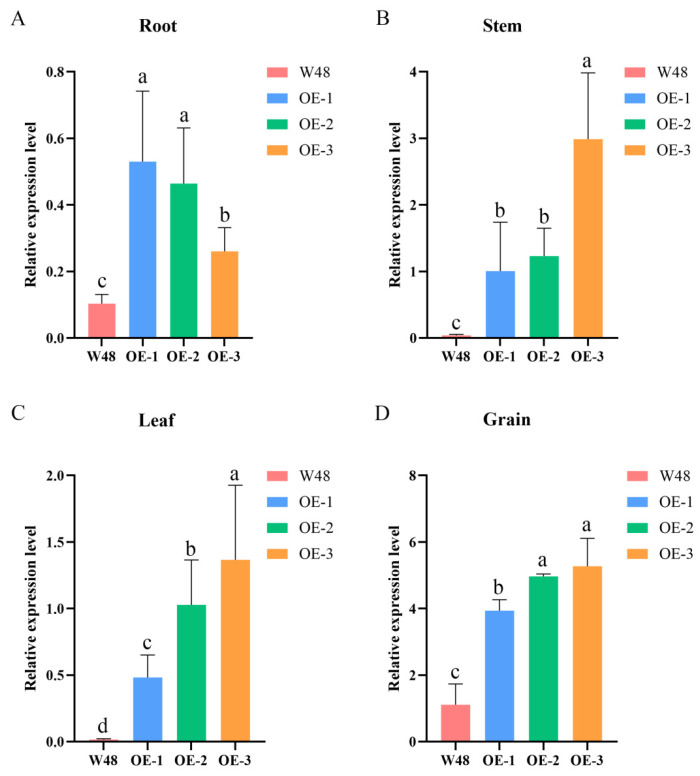
RT-qPCR transcriptional expression of the *TaSBP-A* gene in the roots, stems, leaves, and developing grains at 18 DAF. The data are presented as the mean ± standard deviation (SD) of three biological replicates. Values with different letters differ significantly (*p* < 0.05). (**A**) Root; (**B**) stem; (**C**) leaf; (**D**) grain.

To analyze the expression levels of the *TaSBP-A* gene in different tissues of the three overexpression lines, we conducted a real-time quantitative polymerase chain reaction (RT-qPCR) experiment to detect the transcriptional levels of *TaSBP-A* in the roots, stems, leaves, and grains at 18 days after flowering (DAF) of W48 and transgenic wheat. Compared with W48, the *TaSBP-A* expression levels in roots, stems, leaves, and grains at 18 DAF were significantly higher in the transgenic wheat. Specifically, in roots, the expression levels of OE-1, OE-2, and OE-3 were 5.14-fold, 4.50-fold, and 2.53-fold higher than those of W48, respectively. In stems, the expression levels of OE-1, OE-2, and OE-3 were 25.43-fold, 31.06-fold, and 75.48-fold higher, respectively. In leaves, the expression levels were 29.35-fold, 62.62-fold, and 83.30-fold higher, respectively. In grains, the expression levels of OE-1, OE-2, and OE-3 were 3.54-fold, 4.46-fold, and 4.74-fold higher than those of W48, respectively. It was evident that the expression levels of *TaSBP-A* inOE-1, OE-2, and OE-3 are not consistent. In roots, the expression levels of OE-1, OE-2, and OE-3 decrease sequentially, whereas in stems, leaves, and grains, the expression levels increase sequentially (Figure 1).

### 2.3. Effect of TaSBP-A Overexpression on Major Agronomic Traits of Transgenic Wheats before and after Selenium Application

In transgenic lines and W48, the main agronomic trait changes at the maturity stage were detected (Table 1). When no selenium was applied, the plant height, number of spikelets, and number of grains per spike of the three transgenic lines were significantly higher than those of W48. The plant height increased from 54.06 cm to 64.06 cm, an increase of 18.50%; the number of spikelets increased from 13.2 to 14.75, an increase of 11.74%; and the number of grains in a spike increased from 25.8 to 35, an increase of 35.66%. The spike length and number of tillers also showed an increase, although the differences were not statistically significant. The thousand-grain weight of the transgenic wheat showed a slight reduction compared with W48. Additionally, the grain size analysis showed no significant differences in grain length and width between the three transgenic lines and W48. 

**Figure 2:**
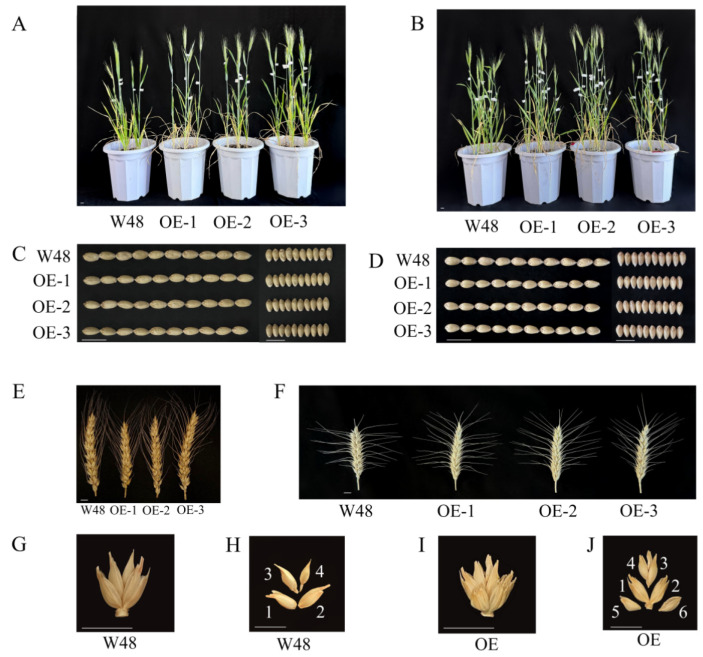
Agronomic trait comparisons of W48 and transgenic lines before and after selenium application. Scale: wheat spikelets and wheat kernels, 1 cm. (**A**) Control wheat at 18 DAF; (**B**) 18-DAF plants after foliar selenium spray treatment; (**C**) length and width of matured kernels in the control group; (**D**) length and width of mature kernels after foliar selenium spray treatment; (**E**) morphology of mature spikes; (**F**) morphology of mature spikes; (**G**,**H**) fertile seeds of W48 basal spikelet; (**I**,**J**) fruiting grains of *TaSBP-A* transgenic basal spikelet.

To examine the impact of additional selenium application, a sodium selenite solution was sprayed on the wheat leaves during the tillering and filling stages. After selenium application, both W48 and transgenic lines in the experimental group showed improvements in plant height, number of tillers, number of spikelets, and number of grains per spike compared with the control group without selenium. Similar to the control group, the main agronomic traits of transgenic wheat in the experimental group, except for thousand-grain weight, were superior to those of W48. In particular, the plant height and number of grains per spike were significantly higher in transgenic wheat than in W48. The plant height increased from 55.24 cm to 67.96 cm, an increase of 23.03%; the number of spikelets increased from 14.33 to 15.86, an increase of 10.68%; and the number of grains in a spike increased from 26.5 to 36.29, an increase of 36.94%. However, the thousand-grain weight of transgenic wheat was lower than that of W48. During the evaluation of agronomic traits, a phenomenon of multiflora was observed in the transgenic wheat across both the control and experimental groups, resulting in an increased number of spikelet seeds at most bases. Specifically, the base number of seeds in a spikelet for W48 was four (Figure 2G,H), while that in the transgenic wheat was six (Figure 2I,J). Moreover, the transgenic wheat showed an additional five and six seeds in the same spikelet compared with W48, indicating a substantial potential increase in yield per spikelet in the genetically modified lines.

### 2.4. Selenium Content Changes in TaSBP-A Transgenic Wheats before and after Selenium Application and Determination of Selenium Form

**Figure 3:**
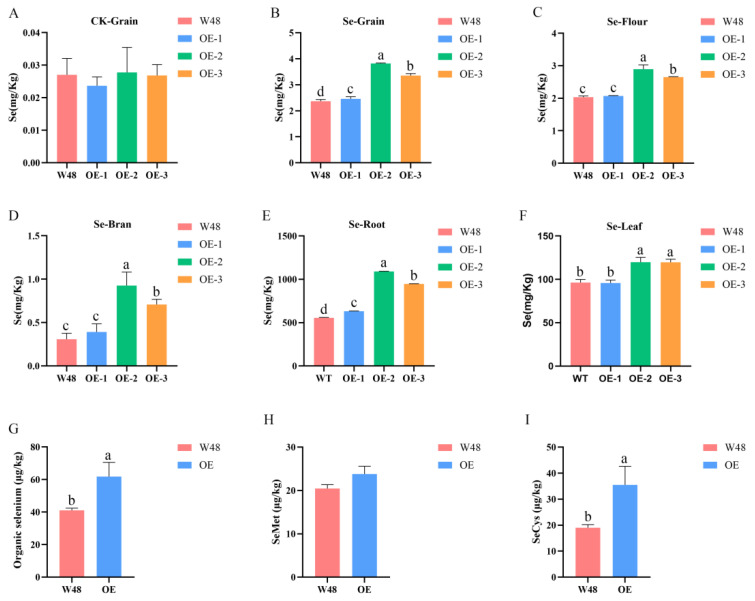
Determination of selenium content and selenium form in W48 and *TaSBP-A* transgenic wheats. (**A**) Selenium content of W48 and transgenic wheat grain without selenium application; (**B**) selenium content of W48 and transgenic wheat grain after selenium application; (**C**) selenium content of W48 and transgenic wheat flour after selenium application; (**D**) selenium content of W48 and transgenic wheat bran after selenium application; (**E**) selenium content of W48 and transgenic wheat roots after selenium application; (**F**) selenium content of leaves of W48 and transgenic wheat after selenium application; (**G**) organic selenium content of W48 and transgenic wheat grain; (**H**) SeMet content of W48 and transgenic wheat grain; (**I**) SeCys content of W48 and transgenic wheat grain. The data are presented as the mean ± SD of three biological replicates. Values with different letters differ significantly (*p* < 0.05).

This research initially quantified the selenium levels in the wheat kernels cultivated under natural conditions, revealing no substantial change in selenium content between transgenic wheat and W48 grains. The selenium concentration in all wheat grain samples was identified within the range of 0.02 to 0.04 mg/kg (Figure 3A), which is significantly below the threshold required for selenium-enriched food products. Additionally, the soil analysis confirmed a deficiency in selenium content, measured at 0.105 mg/kg, thereby classifying the soil as selenium-poor. These results suggest that the selenium enrichment capacity of *TaSBP-A* is not significant at low levels of soil selenium, and thus, additional selenium application may be required to significantly enhance the selenium enrichment function of *TaSBP-A*.

Following selenium application, the examination of selenium content in the roots, leaves, seeds, flour, and bran of W48 and transgenic wheat was conducted through hydroponic and soil culture experiments. The results revealed a notable increase in the selenium levels in the roots, leaves, and seeds of both W48 and transgenic wheat post-selenium application. Moreover, the transgenic wheat exhibited higher selenium levels than W48, with OE-2 and OE-3 showing significant disparities when compared with W48 (Figure 3). The selenium content of the transgenic wheat grains was increased by 61.18% compared with W48. From the results of the assessment of selenium content and differences in wheat kernels, flour, and bran, it was found that the selenium in the kernels was mainly present in the flour, which accounted for 75–95% of the total amount; meanwhile, the selenium content in the bran was low.

To explore the principal selenium species in wheat grains, the selenium species in selenium-treated W48 and OE-2 grains were examined using high-performance liquid chromatography–inductively coupled plasma mass spectrometry (HPLC-ICP-MS), revealing the five following detected forms: Se^4+^, Se^6+^, SeMet, SeCys, and Se-methyl selenocysteine (MeSeCys). Notably, the content of Se^4+^, Se^6+^, and MeSeCys was minor, contributing only 3.6–5.5% to the total selenium and thus suggesting a predominant presence of selenium in organic forms within the seeds, comprising 94.5–96.4% of the total selenium. Following the application of selenium, the organic selenium content in the transgenic wheat was recorded at 61.8 μg/kg, markedly surpassing the level of 41.65 μg/kg observed in W48 grains and indicating a significant elevation in the organic selenium levels in transgenic wheat compared with W48 (Figure 3G). Furthermore, the levels of SeMet and SeCys in the grains of the transgenic wheat were substantially higher than those in W48 (Figure 3H,I). However, on analyzing the distribution of SeMet and SeCys within the total organic selenium, the W48 seeds had a higher SeMet concentration, while the transgenic wheat seeds exhibited a higher SeCys content.

### 2.5. Analysis of Selenium Transport Coefficient in TaSBP-A Transgenic Wheats

**Figure 4:**
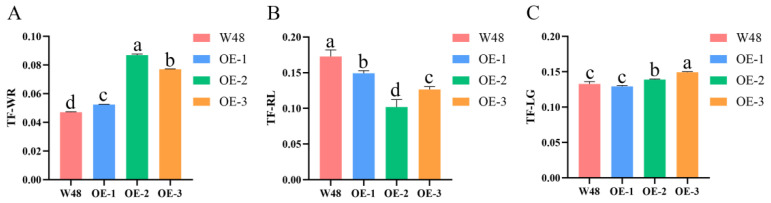
Selenium transport coefficients of W48 and *TaSBP-A* transgenic wheats. (**A**) Migration coefficient of selenium from culture fluid to wheat plants, TF-WR = wheat selenium content/culture fluid selenium content; (**B**) migration coefficient of selenium from root to leaf, TF-RL = leaf selenium content/root selenium content; (**C**) migration coefficient of selenium from flag leaf to seed, TF-LG = leaf selenium content/grain selenium content. The data are presented as the mean ± SD of three biological replicates. Values with different letters differ significantly (*p* < 0.05).

The migration coefficient of an element is indicative of the element’s transport efficiency between different plant tissues, with higher values reflecting stronger transport capabilities. In the context of this research, the selenium transport coefficients were evaluated for W48 and transgenic wheat following selenium supplementation. This assessment entailed an investigation of the selenium transport coefficients from the nutrient solution to the plant and from the roots to the leaves through hydroponic experiments. Subsequently, the selenium transport coefficients from the flag leaves to the seeds were determined using a soil culture experiment. The findings revealed that the selenium migration coefficient from the nutrient solution to the plant was significantly elevated in transgenic wheat in comparison with W48 (Figure 4A). In contrast, the selenium migration coefficient from root to leaf was more pronounced in W48 (Figure 4B). Regarding the selenium migration from the flag leaf to the seed, the results varied between W48 and transgenic wheat. Specifically, OE-2 and OE-3 exhibited significantly higher selenium migration coefficients relative to W48; however, OE-1 showed no significant difference when compared with W48 (Figure 4C). These results indicated that *TaSBP-A* overexpression enhanced the migration of selenium from root to leaf and from flag leaf to grains, which could facilitate plant growth and grain selenium accumulation.

### 2.6. Three-Dimensional Structure Simulation and Molecular Docking of TaSBP-A Protein with Selenite, SeMet, Indole-3-Acetic Acid (IAA), and Gibberellin (GA)

**Figure 5:**
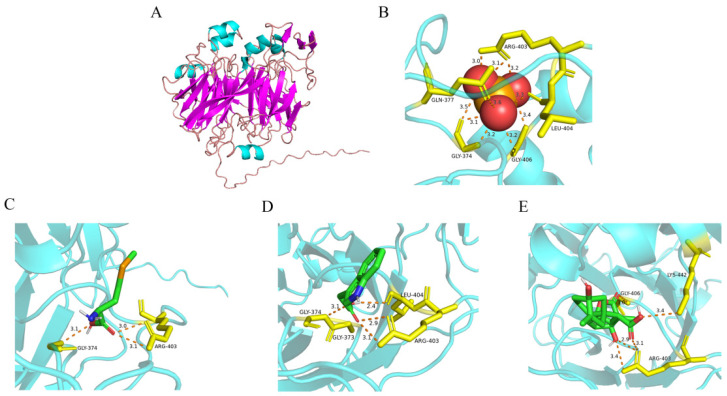
Predicted three-dimensional structure of TaSBP-A protein and its docking simulation with selenite, SeMet, IAA, and GA molecules. (**A**) Three-dimensional structure of TaSBP-A protein; α-helix is indicated in cyan, β-folding in purple, and random curling in reddish brown; (**B**) docking results of TaSBP-A with selenite; (**C**) docking results of TaSBP-A with SeMet; (**D**) docking results of TaSBP-A with IAA; (**E**) docking results of TaSBP-A with GA. Proteins and small molecules mainly rely on hydrogen bonding to connect with each other; the number next to the dotted line indicates the distance between the protein and the small molecule, and the number connected with the amino acid by a short line represents the position of the amino acid.

To elucidate the impact of the three-dimensional structure of the TaSBP-A protein on its functional attributes, an online prediction of the TaSBP-A protein structure was conducted utilizing AlphaFold 2, a state-of-the-art protein structure prediction tool. The prediction process was iterated five times, yielding five potential structural outcomes. The structure with the highest confidence score was then chosen as the definitive model. Subsequently, the selected prediction was visualized using PyMOL, a sophisticated protein structure visualization software. This visualization facilitated the quantitative analysis of the protein’s secondary structural components. The TaSBP-A protein, comprising a total of 495 amino acids, features an α-helix structure that constitutes 8.9% of its composition, predominantly located on the protein’s surface and including a metal-binding site. The β-sheet structure represents 36.0% of the protein, primarily situated at the core, forming a distinct cavity. Lastly, random coils, making up 55.1% of the structure, serve as linkages between the α-helix and β-sheet regions (Figure 5A).

The interaction between TaSBP-A and various small molecules, including selenite, SeMet, IAA, and GA, was investigated through molecular docking simulations using AutoDock. The docking predictions were visualized with the PyMOL software (version 2.2.0). The results revealed that sodium selenite was capable of forming hydrogen bonds with Cys161, Trp97, His160, Ser159, and Ser490 within TaSBP-A (Figure 5B). Similarly, SeMet was observed establishing hydrogen bonds with Arg403 and Gly374 of TaSBP-A (Figure 5C), while IAA formed hydrogen bonds with Gly373, Gly374, Arg403, and Arg404 (Figure 5D). Additionally, GA demonstrated the ability to form hydrogen bonds with Arg403, Gly406, and Lys442 of TaSBP-A (Figure 5E). Upon examining the protein structures, it was observed that the binding sites for these small molecules were situated within pockets or groove structures on the protein surface, comprised of α-helices and random coils.

### 2.7. Transcriptomic Analysis of TaSBP-A Transgenic Wheats

The sequencing data results show that the Q30 values were all above 92%, and the mapped read values ranged between 74 and 83% (Table S1, Supplementary Materials). A breakdown of the total mapped reads aligned to different genomic regions indicates that more than 84% were located in exon regions, fewer than 2.3% were in intron regions, and 8.9–13.49% were in intergenic regions (Table S2). The correlation of gene expression levels between samples can verify the reliability of the experiment and the appropriateness of sample selection, typically requiring the square of the Pearson correlation coefficient between samples to be greater than 0.8. The results showed that apart from a correlation of 0.79 between W48–2 and SBP6–2, all other values were greater than 0.8, demonstrating a high overall precision and good reproducibility between samples (Figure S2).

**Figure 6:**
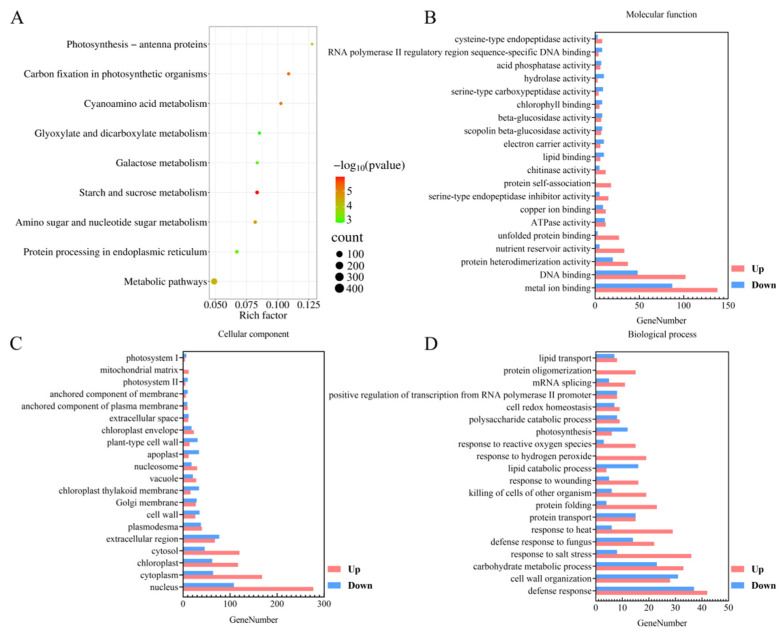
GO enrichment analysis and KEGG enrichment analysis of differentially expressed genes. (**A**) KEGG enrichment analysis; the size of the circle represents the number of differential genes in the pathway, and the color of the bubbles represents the significance level. (**B**–**D**): GO enrichment analysis; pink color indicates genes with up-regulated expression; blue color indicates genes with down-regulated expression.

In order to investigate which gene expression changes were caused by *TaSBP-A* overexpression, the expression of differentially expressed genes in the seeds was analyzed using the seeds of W48 at 18 DAF as the control and the seeds of *TaSBP-A* transgenic wheat OE-3 at 18 DAF as the experimental group. The results showed 3561 differentially expressed genes, including 2206 genes with significantly up-regulated expression and 1355 genes with significantly down-regulated expression. In order to analyze the functions of the differentially expressed genes further, Gene Ontology (GO) enrichment analysis and Kyoto Encyclopedia of Genes and Genomes (KEGG) enrichment analysis were performed on the differentially expressed genes (Figure 6). The KEGG pathway analysis showed that the differentially expressed genes were involved in biological processes, mainly in the metabolic processes of carbon metabolism, metabolic pathways, amino sugar and nucleotide sugar metabolism, starch and sucrose metabolism, amino acid metabolism, metabolism of photosynthesis-related substances, and metabolic processes such as endoplasmic reticulum protein processing (Figure 6A). The results of the GO enrichment analysis showed that the molecular functions were mainly concentrated in metal-ion binding and DNA binding (Figure 6B); the cellular components involved in the differentially expressed genes were primarily the nucleus, cytoplasm, chloroplasts, and cytosol (Figure 6C); and the biological processes involved were mainly defense responses, carbohydrate metabolic processes, salt stress response, response to fungi, response to heat, and protein folding and transporter processes (Figure 6D).

Based on the differential expression of genes in the endosperm of the *TaSBP-A* transgenic wheat and W48 18 DAF and considering the differences in major agronomic traits and seed selenium content between transgenic wheat and W48, a total of 11 genes related to amino acid metabolism and transport, regulation of phytohormones, regulation of gene expression, and plant growth and development were selected. These genes were verified for their expression at the transcriptional level using RT-qPCR (Figure S3).

## 3. Discussion

This study aimed to investigate the effects of overexpressing the *TaSBP-A* gene on wheat growth and grain selenium enrichment. Firstly, the agronomic traits of transgenic wheat and W48 were recorded before and after selenium application. Subsequently, selenium content, selenium forms, and selenium transfer rates in grains were measured. Molecular dynamics simulations of TaSBP-A were used to further explain the reasons for enhanced wheat growth. Previous research on *SBP* genes mainly focused on stress resistance. To analyze changes in gene pathways after overexpressing *TaSBP-A*, differentially expressed genes were identified through transcriptome data.

### 3.1. TaSBP-A Overexpression Enhances Plant Growth and Grain Development

The agronomic data obtained for W48 and *TaSBP-A* transgenic wheat confirmed that *TaSBP-A* influenced wheat throughout its growth and developmental stages (Table 1). Factors such as nutrients, available space, water resources, and phytohormones affect wheat plant height. The research has highlighted that growth hormones and gibberellins play crucial roles in regulating various biological processes, including plant growth and development [31,32,33]. Using AutoDock software (version 1.5.6), the simulations predicted potential docking sites for *TaSBP-A* with growth hormones and gibberellins, suggesting that the increased plant height in *TaSBP-A* transgenic plants could be due to the enhanced synthesis and translocation of these hormones following the gene’s overexpression (Figure 5D,E). Separate studies have shown varied effects of selenium application on wheat. The application of both selenite and selenate reduced the biomass and seed yield across various wheat varieties, regardless of the selenium levels applied [16]. Conversely, selenium application, both via soil and foliar sprays, increased the stem dry weight and seed yield, with seeds from the foliar treatments having a higher organic selenium content than those from soil treatments [28]. The application of sodium selenate to wheat leaves enhanced the photosynthetic rates and carbohydrate metabolism, which in turn increased the seed yield [34]. Similarly, the wheat grown in selenium-rich areas exhibited an increased seed yield and crude protein content [35]. Research into selenium-binding proteins in Arabidopsis and other species has shown that these genes can boost plant resistance to abiotic stresses [26,36,37]. Consistent with previous research findings, our results indicate that foliar application of selenium can promote wheat growth and grain development (Figure 2), improving yield-related traits such as spike length, number of tillers, number of spikelets, and number of grains per spike. Although the thousand-grain weight decreased slightly, it was not significantly different. We hypothesize that the increase in the number of tillers in selenium-treated wheat led to the grains on the tillers being lighter than those on the main spike. Since grain weight was averaged without distinguishing between main and tiller spikes, the overall weight appeared to be reduced. This suggests that while selenium is not essential for plant growth, its application can enhance growth and dry matter accumulation.

### 3.2. TaSBP-A Overexpression Improves Selenium Enrichment in Wheat Grains

Thermodynamic analyses determined that selenate predominated in alkaline and well-oxidized soils (pe + pH > 15); meanwhile, selenite was the primary form in well-drained mineral soils with a pH ranging from acidic to neutral (7.5 < pe + pH < 15) [38]. This suggests that, in the studied soils, selenite was the main form available for plant uptake. Previous research has established that selenate is absorbed through the cell membrane via high-affinity sulfate transporters and is subsequently transported via the xylem as selenium (VI) into the shoots [39,40,41,42]. Conversely, selenite is taken up by the roots through phosphate and silicon transporters [43,44] and is quickly transformed into organic forms of selenium, such as SeMet, MeSeCys, and selenomethionine Se-oxide (SeOMet), which limits its mobility [45]. The elemental selenium is primarily metabolized into its organic state within the chloroplasts or plastids of plants [46]. The metabolic pathway involves the following steps: after being absorbed by the roots, selenate is translocated in its unchanged form to the cells in the above-ground tissues. First, it is reduced to adenosine 5′-phospho-selenite (APSe) by ATP-sulfatase in the chloroplasts, then converted to adenosine 5′-phospho-selenite by glutathione and 5′-phosphoadenosine sulfite reductase. Next, it is reduced to n-divalent selenium ions (Se^2−^) by sulfite reductase [47]. Se^2−^ is converted into SeCys by cysteine synthase [48,49], which is then transformed into SeMet by cystathionine-γ-synthase and cystathionine-β-cleavage enzymes [50]. Finally, SeCys and SeMet can non-specifically replace Cys and Met to form selenium-containing proteins in the chloroplasts and cytoplasm through the action of cysteine-tRNA synthetase and methionine-tRNA synthetase. This study further validated these findings by analyzing the valence states of selenium, specifically Se^4+^, Se^6+^, SeMet, SeCys, and MeSeCys, in wheat grains after the foliar application of selenium. The results showed that SeMet and SeCys were the dominant forms present in the wheat grains, while the levels of Se^4+^, Se^6+^, and MeSeCys were extremely low or undetectable. In addition, the organic selenium content in the grains of both W48 and transgenic wheat exceeded 90% (Figure 3G,I). 

By analyzing the selenium content in the grains of W48 and transgenic wheat, it was found that without selenium fertilizer, there was no significant difference in selenium content between W48 and transgenic wheat grains. Subsequent testing of the soil in the experimental field showed a selenium content of 0.108 mg/kg. Selenium in plants mainly comes from the soil, and the selenium content and forms in the soil directly affect the plant’s absorption capacity. Soil selenium content is classified into five levels: selenium-deficient (<0.125 mg/kg), selenium-poor (0.125–0.175 mg/kg), selenium-adequate (0.175–0.40 mg/kg), selenium-rich (0.40–3.0 mg/kg), and selenium-excessive (>3.0 mg/kg) [51,52]. Therefore, it is speculated that the low selenium content in the environment does not significantly affect plant growth, leading to the possibility that the *TaSBP-A* gene may not significantly enrich selenium. Following the application of foliar selenium, significant increases in the selenium content of wheat grains were observed across all the experimental groups, with transgenic wheat grains showing notably higher selenium levels than those in W48 (Figure 3B). This demonstrates that the foliar application of sodium selenite solution can effectively boost the selenium content in wheat grain; additionally, the *TaSBP-A* gene can enhance wheat’s selenium utilization efficiency. Previous studies have shown that most selenium in wheat grains was retained in the flour and not lost to the bran or paste layer during milling [53], a finding consistent with the selenium distribution observed in this study (Figure 3C,D). Furthermore, it is known that selenite and its metabolites tend to accumulate more than selenate in plant roots, with selenate being more easily distributed from the roots to the stems [54,55]. This was corroborated using hydroponic experiments, which showed that both W48 and transgenic wheat had higher levels of selenium accumulation in their roots compared with their leaves, and transgenic wheat accumulated significantly more selenium than W48 (Figure 3E,F). The tissue-specific expression analysis of *TaSBP-A* indicated that this gene was most highly expressed in roots and seeds [26]. In this study, the three-dimensional structure analysis of TaSBP-A using the AlphaFold 2 platform and molecular docking simulations with sodium selenite and SeMet small molecule ligands identified potential binding sites on TaSBP-A for sodium selenite and SeMet (Figure 5B,C). Due to the high *TaSBP-A* expression and its enhanced capacity to bind free selenium in the roots of transgenic wheat, it was hypothesized that a small portion of sodium selenite absorbed by the leaves could directly bind to TaSBP-A following foliar selenium application. Most of the sodium selenite would then be converted into the organic forms of SeCys and SeMet within the chloroplasts or plastids and would subsequently be bound by TaSBP-A, thereby improving the selenium enrichment in the transgenic wheat grains.

### 3.3. TaSBP-A Overexpression Accelerates Selenium Transport in Wheat Plants

The soil application of selenium facilitates the translocation of selenium from the root system to the aboveground parts of the plant, whereas foliar sprays enable a more direct transfer of selenium from the leaves to the seeds [56]. In cereals like wheat and rice, nodes are crucial for the transport and distribution of mineral elements, such as zinc. These nodes feature complex yet well-organized vascular systems that efficiently deliver minerals to the developing grains [57]. Once the selenium is absorbed by the leaves, it can be directly transported to the grains via the phloem or to adjacent internodes and then redistributed to the grains during the filling and ripening stages. Foliar application introduces selenium into wheat leaves, where it is utilized for daily metabolic needs; any surplus is transformed by the chloroplasts into SeMet and SeCys. These are either incorporated non-specifically in the place of Met and Cys in proteins, stored in vesicles, or converted into volatile dimethyl selenide compounds that are expelled through leaf transpiration. During the irrigation stage, the selenium within vesicles and proteins is reconverted into free SeMet and SeCys. The transportation of sulfate and phosphate across the plasma membranes of root cells occurs against their electrochemical gradients, driven by proton co-transport [58,59], thereby suggesting that selenate and selenite might share this uptake mechanism. The uptake of SeMet is an active, energy-dependent process that is inhibited by respiratory inhibitors such as CCCP and DNP. This uptake requires a selective binding site and metabolic energy, indicating a homodimeric process involving proton (H^+^) transport [60], as supported by the results of earlier studies [61]. SeMet can be transported via non-specific amino acid transporters (AATs) for neutral amino acids. The presence of amino acids such as methionine, tyrosine, phenylalanine, leucine, serine, alanine, valine, proline, threonine, cysteine, and glutamine significantly hampers SeMet uptake. In a previous study, the expression of the *TaSBP-A* gene was investigated across ten developmental stages of the endosperm in spring wheat in China, revealing a high level of consistency with the endosperm’s developmental process [62]. During the irrigation stage, as the nutrients are actively transported to the grains, selenium is also transported in the organic forms of SeMet and SeCys. 

**Figure 7:**
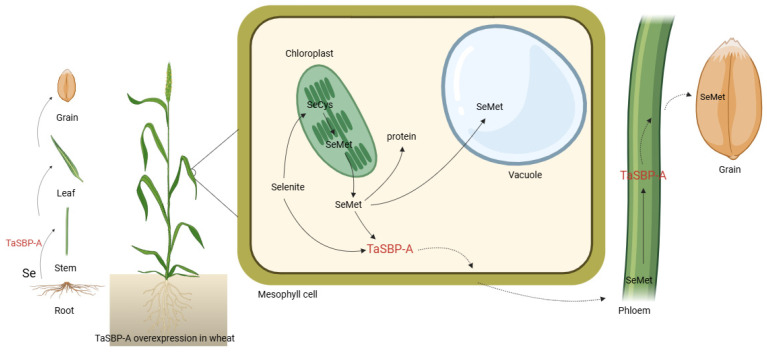
Schematic diagram of TaSBP-A binding and transportation of selenium.

In the transgenic wheat, the levels of SeMet and SeCys in the grains were significantly higher than those in W48 (Figure 3G,I). TaSBP-A, a cytoplasmic protein with a transport function, was hypothesized to enhance the transport efficiency of both SeMet and SeCys as its expression increases, thereby boosting the selenium content in the grains. A comparative analysis of selenium transport coefficients from leaves to seeds under the foliar application of sodium selenite revealed that the transport capacity of transgenic wheat for selenium exceeded that of W48. However, the migration coefficient of OE-1 did not differ significantly from W48 (Figure 4C). The examination of *TaSBP-A* expression in flag leaves and seeds of W48 and transgenic wheat 18 days post-anthesis showed increasing expression levels in OE-1, OE-2, and OE-3 (Figure 1). OE-1, with the lowest overexpression level, might consequently have a lower selenium uptake and translocation from leaves to grains compared with OE-2 and OE-3, whose translocation coefficients increased with the rising gene expression. Additionally, this study identified fourteen differentially expressed genes involved in oxidative phosphorylation, thirty in amino acid transport, and three with neutral amino acid transmembrane transporter protein activity through transcriptome analysis (Table S3). Thus, the overexpression of *TaSBP-A* significantly enhances the ability of transgenic wheat to bind and transport selenium in both leaves and grains compared with W48. Following the foliar application of sodium selenite solution, the selenium content, enriched by the action of *TaSBP-A* within the chloroplasts, increases as the grains enter the filling stage. Selenium is then transported to the grains via the phloem or neighboring internodes, resulting in an increase in grain selenium content. To illustrate these processes, a pathway diagram was created to visually represent the mechanism of selenium uptake and distribution in transgenic wheat, as shown in Figure 7.

### 3.4. Transcriptional Regulation Networks of TaSBP-A Involved in Selenium Accumulation in Wheat Grains

In this study, 11 genes associated with phytohormone regulation, amino acid metabolism, transport, and regulatory gene expression were selected from differentially expressed genes for validation at the transcript level (Figure S2). Through this selection, we aimed to explore the effects of *TaSBP-A* overexpression on wheat growth and development. The transcriptome data revealed that the expression of all these genes was up-regulated in transgenic wheat, likely contributing to the enhanced plant growth. The following were identified as noteworthy: the Golgi SNAP receptor complex, involved in protein transport between the endoplasmic reticulum and the Golgi apparatus [63]; the amino acid transporter ANT1-like, responsible for transporting central and aromatic amino acids; and the amino acid permease 6-like, a membrane-bound protein engaged in amino acid transmembrane transport [64]. Additionally, in amino acid metabolism, the aminotransferase-related protein facilitates the transfer of amino groups between amino acids and keto acids. The elongator complex protein, which affects RNA transcription and gene expression, also plays a role in regulating abscisic acid (ABA) signaling and plant growth and development [65]. The expression analysis results indicated that the expression of genes involved in amino acid metabolism and transport and those associated with hormone regulation was significantly higher in transgenic wheat than in W48. We speculate that, following *TaSBP-A* overexpression during the grain filling stage, selenomethionine and selenocysteine are released from vesicles or selenoproteins, leading to up-regulated gene expression. This up-regulation affects amino acid trans-membrane transport and enhances selenium transport and enrichment in the grains. 

Serine/threonine protein kinases and MOB kinases are essential for maintaining stem cell differentiation, regulating the cell cycle, and promoting cell growth. PP1 is a member of the serine/threonine protein phosphatase family. In Arabidopsis, a point mutation in the PIPE gene (phosphatase-induced pleiotropic effect), a member of this gene family, leads to extreme dwarfism and abnormal cell morphology, highlighting its significant role in plant development. Another member of this family, PP2, is involved in the regulation of growth and development and acts as a negative regulator in hormone signaling pathways triggered by cold and drought conditions [66,67]. An interaction between PIPE and the DALL3 gene has been suggested, with DALL3 being a crucial gene in the regulation of intracellular GA synthesis. Yeast two-hybrid and bimolecular fluorescence assays have shown an interaction between AtSBP1 and AtDALL3 [68], leading to the hypothesis that *TaSBP-A* may interact with the PIPE and DALL3 gene expression pathways. As upstream genes, PIPE and DALL3 are posited to regulate the GA synthesis pathway, thereby increasing GA content within tissues and affecting the growth and development of wheat plants. Isopentenyl-diphosphate Delta-isomerase I-like plays a key role in regulating plant growth and development, participating in hormonal regulation and the plant’s response to abiotic stress [69,70]. 

The WRKY family of transcription factors contributes to a wide range of biological processes, including the regulation of growth and development, fruit ripening, and resilience against biotic and abiotic stresses [71]. CiWRKY40–4 was identified as a factor that delayed leaf senescence in *Arabidopsis* [72], and an interaction exists between WRKY and MAPK proteins. This interaction can facilitate the linkage of upstream and downstream receptors with target genes via phosphorylated signaling cascades, thereby influencing gene expression [73]. Phytochrome-interacting ankyrin-repeat proteins belong to a group of transcription factors that engage with photosensitive pigment proteins, playing a crucial role in various signaling pathways to control plant growth and developmental processes [74]. GAPCP1, a glyceraldehyde-3-phosphate dehydrogenase, is involved in the oxidation and phosphorylation of glyceraldehyde 3-phosphate and plays a critical role in sugar metabolism. The up-regulation of genes related to photosynthesis, carbohydrate metabolism, and polysaccharide catabolism after *TaSBP-A* overexpression may, to a certain extent, increase the efficiency of photosynthesis and promote the synthesis and metabolism of nutrients in transgenic wheat, thereby promoting plant growth, grain development, and increasing yield and biomass. *TaSBP-A* was overexpressed in transgenic wheat roots, stems, leaves, and grains, and the regulation of many genes occurred throughout the growth process, ultimately causing improved wheat agronomic traits, selenium transport efficiency, and grain selenium accumulation.

## 4. Materials and Methods

### 4.1. Wheat Materials, Culture Methods, and Sodium Selenite Treatment 

The experimental materials were *TaSBP-A* overexpressing lines (OE-1, OE-2, OE-3) obtained from wheat variety W48 as the genetic transformation recipient (W48 was provided by the Institute of Crop Science, the Chinese Academy of Agricultural Sciences, Beijing, China) [26]. W48 was derived from crossing between Fielder and CB037, which have resistance to powdery mildew, high transformation efficiency, and middle grain quality. 

For greenhouse cultivation, the plants were grown in pots, with five plants per pot, and watered once every three days using equal amounts of water. The pots used had an outer diameter of 27.5 cm, an inner diameter of 23 cm and a height of 25 cm. The soil used was a 2:1 mix of Danish Pindstrup peat (purchased from Pindstrup Peat, Ryomgaard, Denmark) and vermiculite. Plant growth was supplemented with plant filler lights (8.6 Klux), simulating 14 h of daylight and 10 h of nighttime while maintaining air humidity around 60%. Upon entering the tillering stage, the plants were randomly divided into a control group and an experimental group, with 12 pots in each group. The experimental group received the following treatments: (1) When the plants were at the tillering stage, 5 mL of 0.5% sodium selenite solution was sprayed on the leaves of each pot, and watering was withheld for three days after spraying; (2) When more than half of the wheat plants reached the flowering stage, another 5 mL of 0.5% sodium selenite solution was sprayed on the flag leaves and the parts below them, avoiding wetting the spikes. 

In the hydroponic treatment, the transgenic wheat seedlings were cultured in half-Hoagland nutrient solution [75] for three days and then replaced with the Hoagland nutrient solution after three days. The nutrient solution was replaced every three days. When the seedlings entered the three-leaf and one-heart stage, they were cultured in Hoagland nutrient solution containing 0.5% sodium selenite, and the culture solution was changed every three days for seven days.

### 4.2. Positive Identification of Transgenic Wheat

Seven leaves and seven mature grains were randomly selected from each of the three transgenic wheat lines, along with one leaf and one mature grain from W48 wheat, for homozygosity identification. The results of the experiment were repeated three times. Genomic DNA was extracted from transgenic wheat leaves using the 2 × CTAB method [76] and detected by PCR using detection primers designed according to the NOS terminator sequences downstream of the *TaSBP-A* gene and the vector pWMB110 (Table S4). The 2 × CTAB extraction buffer was purchased from Solarbio, Beijing, China. Chloroform with a purity of ≥99.8%, isoamyl alcohol with a purity of ≥98.5%, and anhydrous ethanol with a purity of ≥99.7% were used. Additionally, PAT/bar test strips (purchased from Shanghai Yulong Biotechnology Co., Ltd., Shanghai, China) were utilized to test the seeds of the transgenic wheat, following the instructions provided in the manual.

### 4.3. RNA Extraction, cDNA Synthesis, PCR, and Real-Time Quantitative Reverse RT-qPCR

Total RNA was extracted from roots, stems, leaves, and 18-day post-flowering seeds of wheat using an RNA extraction kit, reverse transcribed and synthesized into cDNA using a reverse transcription kit, and amplified by PCR using high-fidelity DNA polymerase. The transcript levels of the target genes were detected by RT-qPCR using the wheat UBI gene as an internal reference (Table S5). RNA extraction kit was purchased from Takara Bio, Kusatsu, Japan; reverse transcription reagent and RT-qPCR reagent were purchased from Vazyme, Nanjing, China. The centrifuge and PCR instrument were purchased from Eppendorf, Hamburg, Germany; the water bath was purchased from Beijing Zhongkeer Instrument Co., Ltd., Beijing, China; and the RT-qPCR instrument was purchased from Bio-Rad, Hercules, CA, USA.

### 4.4. Determination of Major Agronomic Traits in Mature Wheat

When the wheat in the greenhouse matured, the whole plant was collected, and the main agronomic traits were measured: plant height, main spike length, number of tillers, number of spikelets, number of grains, number of kernels in the main spike, and 1000-grain weight. Plant height was measured from the bottom of the plant to the top of the main spike, and spike length was measured from the bottom spike to the top spike, excluding the length of the awn. The number of tillers was counted as effective tillers, i.e., branches with developed spikes. Thousand-grain weight was counted by grabbing a handful of kernels with the hand, weighing them, and then calculating the weight of one thousand grains by repeating the process three times. The measuring tools used were tape measure and vernier caliper. The balance was purchased from Sartorius Scientific Instruments, Beijing, China.

### 4.5. Measurement of Selenium Content and Selenium Mobility Coefficient

The elemental content of selenium was determined using ICP-MS. A certain amount of the powder sample was weighed and placed in a polytetrafluoroethylene ablation tank, and 5 mL of nitric acid was added. After the reaction, the sample was covered, sealed, and placed in the microwave ablator. The specific ablation steps are shown in Table S6, and the ablation parameters are shown in Table S7. After the temperature was cooled to less than 50 °C, the ablator jar was removed from the microwave ablator and put into a fume cupboard. The orifice of the ablator jar tube was opened slowly to release the gas; then, it was entirely opened after the pressure in the tube was equilibrated with that outside of the tube. Then, all liquids were rinsed using ultrapure water and transferred to a 25 mL volumetric flask in total. All the liquid was transferred to a 25 mL volumetric flask, rinsed four times, and diluted and fixed with ultrapure water on the scale to be measured. The electronic balance was purchased from Shanghai Sunny Hengping Scientific Instruments Co., Ltd., Shanghai, China, model Sunny Hengping FA-1004; the microwave digestion system was purchased from MILESTONE, model ETHOS 1; the inductively coupled plasma mass spectrometer was purchased from Thermo Fisher Scientific, Waltham, MA, USA, model iCAPQ; nitric acid was purchased from Sinopharm Chemical Reagent Co., Ltd., Shanghai, China; and the elemental standard solutions were purchased from the National Nonferrous Metals and Electronic Materials Analysis and Testing Center, Beijing, China. The blank control was treated in the same way. The data obtained using ICP-MS were processed using the following formula, and the units were standardized as follows:

Calculation formula: $W=\frac{\left(C-C_{0}\right)\times V\times N}{m}$

W: The content of the target substance in the specimen in mg/kg;

C: Concentration of the target substance in the test solution in mg/L;

C_0_: Concentration of target in blank control in mg/L;

V: Volume of the finalized volume in mL;

N: dilution factor;

M: Sample size of the specimen in g.

The culture fluid–root migration coefficient (TF-WR), which is the ratio of the selenium content in the roots to the selenium content of the culture fluid, was used to measure the ability of the wheat root system to absorb selenium from the nutrient solution. The root–leaf transport coefficient (TF-RL), which is the ratio of selenium content in leaves to selenium content in roots, was used to assess the efficiency of selenium transfer from roots to leaves. The flag leaf–seed migration coefficient (TF-LG), which is the ratio of selenium content in the seed to the selenium content in the flag leaf, was used to assess the efficiency of selenium transfer from the flag leaf to the seed. The results were plotted using GraphPad Prism 8 software and analyzed for significance.

### 4.6. Three-Dimensional Structure Prediction and Molecular Docking Simulation

The amino acid sequence of TaSBP-A was located and downloaded in the Ensemble plant (http://plants.ensembl.org/index.html (accessed on 26 May 2024)), and the protein structure was predicted by logging into the AlphaFold 2 online website. The simulation was performed for five cycles. The output of the results was the parameters, such as the confidence score for each residue, the number of matching template sequences, the prediction alignment error, and five different levels of three-dimensional protein models. The prediction results were in PDB format files, and the protein 3D structures in the PDB files were visualized using the PyMOL software. The molecular structures of selenomethionine, growth hormone, and gibberellin were located and downloaded in SDF file format from the small molecule structure online search site PubChem (https://pubchem.ncbi.nlm.nih.gov/ (accessed on 6 May 2024)). The highest-scoring protein structure model in the AlphaFold 2 prediction results was selected, and the AutoDock software was used to simulate the docking between TaSBP-A and small molecules, selecting the highest-scoring prediction result, and using PyMOL software to visualize and label the results.

### 4.7. Transcriptome Sequencing and Data Analysis

The developing grains at 18 DAF of W48 and three *TaSBP-A* transgenic pure lines were obtained, quickly peeled off the glumes, and then placed in a 15 mL centrifuge tube immersed in liquid nitrogen for rapid freezing; the seeds weighed about 10 g. The seeds were sent to Suzhou Jinwei Zhi Biotechnology Co., Suzhou, China, to complete the construction of the mRNA library and sequencing work. To improve the data quality, the pass filter data in fastq format were processed using Cutadapt (V1.9.1; third cutoff: 20; error rate: 0.1; splice overlap: 1 bp; minimum length: 75; and proportion of N: 0.1). The reference genome sequences and gene model annotation files of related species were downloaded from genome websites such as UCSC, NCBI, and ENSEMBL. Hisat2 (v2.0.1) was used to index the reference genome sequences, and finally, the clean data were aligned to the reference genome using the software Hisat2 (v2.0.1). Fast-format transcripts were converted from the known gff annotation file, which was used as the reference gene file, and HTSeq (v0.6.1) was used as the basis for estimating gene and expression levels. Differential expression analysis was performed using DESeq2 Bioconductor, a model based on negative binomial distribution with the Padj of genes set to <0.05 to detect the differentially expressed genes.

GO [77] contains three ontologies that describe the molecular function, cellular component, and biological process of differentially expressed genes. The screening criterion for significant enrichment is over-represented—value ≤ 0.05. In organisms, different genes need to coordinate with each other to perform their biological functions, and the KEGG [78] analyzes the metabolic pathways to identify the differences in the differentially expressed genes compared with the whole genome. KEGG also analyzes metabolic pathways to discover pathways significantly enriched in differentially expressed genes compared with the genome-wide background. The screening criteria for significant enrichment are Q—value ≤ 0.05.

### 4.8. Statistical Analysis of Data

Data were analyzed using one-way analysis of variance (ANOVA), and means were compared by Duncan’s multiple range tests using IBM SPSS Statistics 26 software (SPSS Inc., Chicago, IL, USA). Graphs were established by GraphPad Prism 8 software. Different letters indicate significant differences (*p* < 0.05).

## 5. Conclusions

*TaSBP-A* overexpression significantly improved wheat growth and grain development, including an increased tiller count, plant height, spike length, spikelet number, and grain number. Under the growth conditions of selenium-poor soil, the overexpression of *TaSBP-A* had no clear selenium-enrichment effects in the grains. However, following the application of sodium selenite via foliar spraying, *TaSBP-A* overexpression significantly increased the grain selenium content; meanwhile, plant height, spike length, spikelet count, and grain number were all notably improved. The three-dimensional structure simulation and molecular docking of the TaSBP-A protein showed potential binding sites on TaSBP-A for sodium selenite and SeMet, which featured pockets and grooves composed of α-helices and random coils, facilitating stable selenium binding and translocation. Translocation efficiency analysis indicated that *TaSBP-A* overexpression accelerated selenium translocation from culture solution to roots and from flag leaf to grains, ultimately enhancing grain selenium accumulation. The results indicate that the *TaSBP-A* gene has the potential to improve selenium content in wheat grains through biofortification. This provides a candidate gene and theoretical basis for selenium biofortification in wheat, contributing to addressing the global issue of selenium deficiency in human diets. 

## Figures and Tables

**Table 1:** The main agronomic trait changes in W48 and *TaSBP-A* transgenic wheat *.

Materials	Plant Height (cm)	Spike Length (cm)	Tiller Number	Spikelet Number	Kernel per Spike	Thousand-Grain Weight (g)
CK-W48	54.06 ± 4.29 ^d^	7.68 ± 0.59	1.00 ± 0.01 ^b^	13.2 ± 1.48 ^c^	25.8 ± 6.30 ^b^	36.84 ± 0.58 ^a^
CK-OE-1	62.74 ± 5.22 ^bc^	7.7 ± 0.24	1.00 ± 0.01 ^b^	14.6 ± 1.14 ^abc^	33.6 ± 3.21 ^a^	31.11 ± 0.10 ^cd^
CK-OE-2	60.52 ± 3.49 ^c^	7.65 ± 0.17	1.00 ± 0.01 ^b^	13.6 ± 0.55 ^bc^	34.4 ± 5.86 ^a^	33.33 ± 1.54 ^b^
CK-OE-3	64.06 ± 2.20 ^abc^	7.98 ± 0.42	1.5 ± 0.58 ^ab^	14.75 ± 0.96 ^abc^	35 ± 2.94 ^a^	33.69 ± 1.36 ^b^
Se-W48	55.24 ± 2.77 ^d^	7.93 ± 0.77	1.3 ± 0.48 ^ab^	14.33 ± 1.75 ^abc^	26.5 ± 3.56 ^b^	33.67 ± 0.61 ^b^
Se-OE-1	66.7 ± 2.38 ^ab^	8.14 ± 0.56	1.8 ± 0.42 ^a^	15.86 ± 2.04 ^a^	33.86 ± 6.15 ^a^	29.67 ± 1.19 ^d^
Se-OE-2	67.96 ± 1.53 ^a^	8.01 ± 0.50	1.8 ± 0.42 ^a^	15.43 ± 1.51 ^ab^	36.29 ± 4.57 ^a^	32.16 ± 1.36 ^bc^
Se-OE-3	66.86 ± 2.85 ^ab^	8.03 ± 0.61	1.67 ± 0.52 ^a^	15.33 ± 1.63 ^ab^	36.2 ± 4.27 ^a^	32.53 ± 0.66 ^bc^

* Data are mean ± SD. Values with different letters in the same column differ significantly (*p* < 0.05). CK indicates the control group without selenium application, and Se indicates the experimental group with selenium application.

## Data Availability

Data are contained within the article.

## Supplementary Materials

The following supporting information can be downloaded at: https://www.mdpi.com/article/10.3390/ijms25137007/s1.

## Abbreviations

ABA	Abscisic acid
GA	Gibberellins
GO	Gene Ontology
IAA	Indoleacetic acid
ICP-MS	Inductively coupled plasma mass spectrometry
KEGG	Kyoto Encyclopedia of Genes and Genomes
PCR	Polymerase chain reaction
ROS	Reactive oxygen species
RT-qPCR	Real-time quantitative polymerase chain reaction
SBP	Selenium-Binding Protein
Se	Selenium
SeCys	Selenocysteine
SeMet	Selenomethionine
SeOMet	Selenomethionine Se-oxide
MeSeCys	Se-methyl selenocysteine
