# Supplementary material for: Overexpression of Wheat Selenium-Binding Protein Gene TaSBP-A Enhances Plant Growth and Grain Selenium Accumulation under Spraying Sodium Selenite
Source: Int J Mol Sci. 2024 Jun 26;25(13):7007. doi: 10.3390/ijms25137007 (PMC11240915; doi:10.3390/ijms25137007)
Supplement: Supplementary file 1 [file ijms-25-07007-s001.zip › ijms-3071995-supplementary.pdf]

Table S1. Sequencing data quality statistics of transfer group.

Sample	Raw Reads	Clean Reads	Q20 (%)	Q30 (%)	GC (%)	Total Mapped Reads（%）
W48-1	56571782	55771474	97.63	93.64	52.57	82.34
W48-2	41072702	40199596	97.18	92.75	52.01	74.12
W48-3	44534024	43788628	97.23	92.84	52.04	75.86
SBP6-1	55169558	54397866	97.37	93.17	51.90	81.24
SBP6-2	53665364	53151316	97.02	92.38	51.80	79.73
SBP6-3	42298656	41715788	97.49	93.34	52.07	81.68

Table S2. Distribution of Reads in different regions of the reference genome.

Sample	Exon（%）	Intergenic（%）	Intron（%）
W48-1	84.21	13.49	2.3
W48-2	88.74	9.84	1.42
W48-3	88.33	10.13	1.54
SBP6-1	88.42	9.99	1.59
SBP6-2	89.65	8.93	1.42
SBP6-3	87.4	10.84	1.76

Table S3. Biological processes (BP) or pathways in which differentially expressed genes are involved or their molecular functions (MF).

Gene ID	BP or MF or Pathway
TraesCS1B02G148500	ko00190 Oxidative phosphorylation;ko01100 Metabolic pathways;
TraesCS6A02G097000	ko00190 Oxidative phosphorylation;<br>ko00860 Porphyrin and chlorophyll metabolism;<br>ko01100 Metabolic pathways;<br>ko01110 Biosynthesis of secondary metabolites;<br>ko01240 Biosynthesis of cofactors;
TraesCS2D02G521600	ko00190 Oxidative phosphorylation;<br>ko01100 Metabolic pathways;<br>ko04145 Phagosome;
TraesCS1B02G369000	ko00190 Oxidative phosphorylation;<br>ko01100 Metabolic pathways;
TraesCS6A02G186300	ko00190 Oxidative phosphorylation;<br>ko01100 Metabolic pathways;<br>ko04145 Phagosome;
TraesCS2D02G503000	ko00190 Oxidative phosphorylation;<br>ko01100 Metabolic pathways;
TraesCS1B02G124600	ko00190 Oxidative phosphorylation;
TraesCS1B02G049200	ko00190 Oxidative phosphorylation;
TraesCS3D02G539900	ko00190 Oxidative phosphorylation;
TraesCS3D02G540100	ko00190 Oxidative phosphorylation;
TraesCS6A02G146600	ko00190 Oxidative phosphorylation;<br>ko01100 Metabolic pathways;<br>ko04145 Phagosome;

TraesCS3A02G534700	ko00190 Oxidative phosphorylation;
TraesCS1A02G273500	ko00190 Oxidative phosphorylation;
TraesCS2D02G587100	ko00190 Oxidative phosphorylation;
TraesCS5D02G180000	GO:0015171:amino acid transmembrane transporter activity;<br>GO:0015173:aromatic amino acid transmembrane transporter activity;<br>GO:0015175:neutral amino acid transmembrane transporter activity;
TraesCS2B02G266600	GO:0015172:acidic amino acid transmembrane transporter activity;<br>GO:0015171:amino acid transmembrane transporter activity;<br>GO:0015175:neutral amino acid transmembrane transporter activity;<br>GO:0015293:symporter activity;
TraesCS5B02G173000	GO:0015171:amino acid transmembrane transporter activity;<br>GO:0015173:aromatic amino acid transmembrane transporter activity;<br>GO:0015175:neutral amino acid transmembrane transporter activity;
TraesCS1B02G132900	GO:0000045:autophagosome assembly;<br>GO:0000422:mitochondrion degradation;<br>GO:0006497:protein lipidation;<br>GO:0034497:protein localization to pre-autophagosomal structure;<br>GO:0015031:protein transport;
TraesCS2D02G520100	GO:0000045:autophagosome assembly;<br>GO:0015031:protein transport;
TraesCS5A02G391000	GO:0006875:cellular metal ion homeostasis;<br>GO:0055072:iron ion homeostasis;<br>GO:0007275:multicellular organismal development;<br>GO:0015031:protein transport;<br>GO:1990388:xylem-to-phloem iron transport;
TraesCS6A02G088900	GO:0006888:ER to Golgi vesicle-mediated transport;<br>GO:0015031:protein transport;<br>GO:0048209:regulation of vesicle targeting, to, from or within Golgi;<br>GO:0009737:response to abscisic acid;<br>GO:0006906:vesicle fusion;
TraesCS5D02G119700	GO:0006897:endocytosis;<br>GO:0015031:protein transport;<br>GO:0016192:vesicle-mediated transport;
TraesCS6A02G050300	GO:0006914:autophagy;<br>GO:0015031:protein transport;
TraesCS6A02G133300	GO:0007049:cell cycle;<br>GO:0007032:endosome organization;<br>GO:0055075:potassium ion homeostasis;<br>GO:0015031:protein transport;<br>GO:0055078:sodium ion homeostasis;<br>GO:0010091:trichome branching;<br>GO:0007033:vacuole organization;<br>GO:0016192:vesicle-mediated transport;
TraesCS6A02G097100	GO:0015031:protein transport;

TraesCS2D02G506400	GO:0015031:protein transport;
TraesCS4B02G003400	GO:0015031:protein transport;
TraesCS2D02G574200	GO:0015031:protein transport;
TraesCS5D02G277900	GO:0015031:protein transport;
TraesCS5B02G270200	GO:0015031:protein transport;
TraesCS5A02G269800	GO:0015031:protein transport;
TraesCS7A02G445600	GO:0015031:protein transport;
TraesCS6A02G193500	GO:0015031:protein transport;<br>GO:0006904:vesicle docking involved in exocytosis;
TraesCS1B02G008200	GO:0015031:protein transport;<br>GO:0007034:vacuolar transport;
TraesCS1D02G002600	GO:0015031:protein transport;<br>GO:0007034:vacuolar transport;
TraesCS5D02G048100	GO:0015031:protein transport;<br>GO:0007264:small GTPase mediated signal transduction;<br>GO:0016192:vesicle-mediated transport;
TraesCS5B02G042400	GO:0015031:protein transport;<br>GO:0007264:small GTPase mediated signal transduction;<br>GO:0016192:vesicle-mediated transport;
TraesCS6A02G113800	GO:0015031:protein transport;<br>GO:0016192:vesicle-mediated transport;
TraesCS6A02G086900	GO:0015031:protein transport;<br>GO:0016192:vesicle-mediated transport;
TraesCS6A02G168000	GO:0015031:protein transport;<br>GO:0032012:regulation of ARF protein signal transduction;<br>GO:0016192:vesicle-mediated transport;
TraesCS5A02G259200	GO:0032456:endocytic recycling;<br>GO:0006896:Golgi to vacuole transport;<br>GO:0009860:pollen tube growth;<br>GO:0015031:protein transport;<br>GO:0042147:retrograde transport, endosome to Golgi;<br>GO:0019953:sexual reproduction;
TraesCS3A02G392800	GO:0042938:dipeptide transport;<br>GO:0009860:pollen tube growth;<br>GO:0015031:protein transport;
TraesCS3D02G385600	GO:0042938:dipeptide transport;<br>GO:0009860:pollen tube growth;<br>GO:0015031:protein transport;
TraesCS6A02G175000	GO:0051028:mRNA transport;<br>GO:0006999:nuclear pore organization;<br>GO:0015031:protein transport;
TraesCS1B02G051100	GO:0051028:mRNA transport;<br>GO:0015031:protein transport;
TraesCS1B02G041300	GO:0051028:mRNA transport;

TraesCS1B02G057100	GO:0015031:protein transport;<br>GO:0000972:transcription-dependent tethering of RNA polymerase II gene DNA at nuclear periphery;<br>GO:0090114:COPII-coated vesicle budding;<br>GO:0051028:mRNA transport;<br>GO:1904263:positive regulation of TORC1 signaling;<br>GO:0015031:protein transport;

Table S4. Sequences of primes used for selection of wheat transgenic lines.

Primer name(s)	Primer sequences (5′-3′)
TaSBP-A-F	ATGGCCGCAGCGGTGGCTGA
110-NOS terminator-R	TAATTGCGGGACTCTAATC

Table S5. RT-qPCR primer.

Gene	F-terminal primer	R-terminal primer
Ubi	TGACACCATCGACAACGTGA	GAGGGTGGACTCCTTCTGGA
TaSBP-A	AGCACAGGTCTTCTTCCA	CCAAGTAGAGATAACGGTCAT
amino acid transporter ANT1-like	CGGGGATTTGGGTCAGAAGT	AGCCAAGTATCCGCACACTC
amino acid permease 6-like	ATCGGACGGTCGAATTGCTT	GCTGGCAGAAGACCTGGTAG
aminotransferase-related protein	TATGCTCACCGAACAACCCC	CTTCTTGCTTCTCACAGCGC
MOB	GCAACCCTCGGTAGTGGAAA	GGAGGGGAGCCAGTTCTTTC
serine/threonine-protein phosphatase	GCAAGAGGAGGTTCAATGTTCG	CTTTTTACCCGCGATGTGCC
Golgi SNAP receptor complex	TCCCAAAGGAACGTGTGAGG	TTCGCACCACCACTTGACAT
elongator complex protein	GAGGAGTTGTGCAGTGGCT	AAGGTCCTGGATGCGCTTC
WRKY	ATGCCCTTCACAGAGCGTTC	TGCTCAACATGATCCCACCA
isopentenyl-diphosphate Delta-isomerase I-like	ATGGACACATGTCGCAGTTA	TCCTCTGCGATGTACTGCTG
phytochrome-interacting ankyrin-repeat protein	CGTCTCCTGAATGAAGGGATGG	ATGCGCTTCATCTCGTACGC
GAPCP1	TGAAGGGATGGATGTCAATGCA	GCTTCATCTCGTACGCCCA

Table S6. ICP-MS digestion steps.

Steps	Temperature(℃)	Holding time(min)
1	100	3
2	140	3
3	160	3
4	180	3
5	190	15

Table S7. ICP-MS Instrument Parameters.

ICP-MS Parameter	Value
RF power	1550 W
Pump Speed	40 rpm
S/C temperature	2.7℃
Smpl Depth	5 mm
Cool flow	14 L/min
Auxilliary flow	0.8 L/min
Nebulizer flow	1.122 L/min

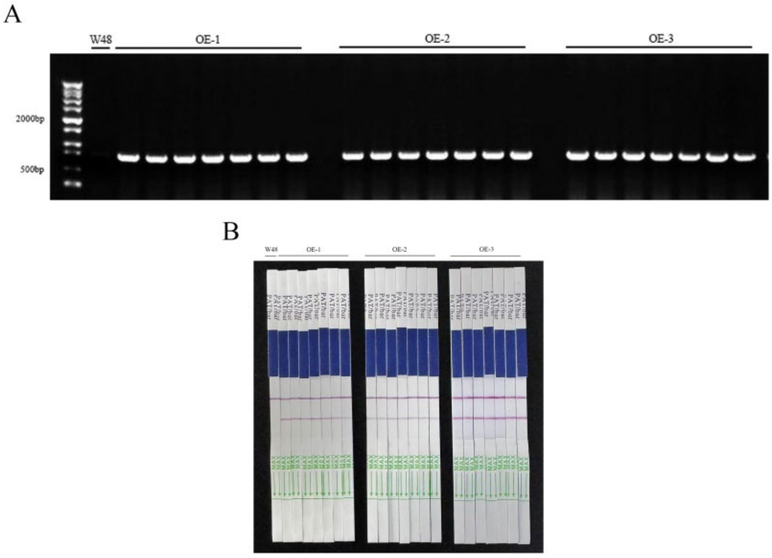

Figure S1. Identification of *TaSBP-A* transgenic wheat positive plants.

（A）Genomic PCR of W48 and three overexpression lines OE-1, OE-2, and OE-3; （B）PAT/bar test strip assay for W48 and three overexpression lines OE-1, OE-2, and OE-3.

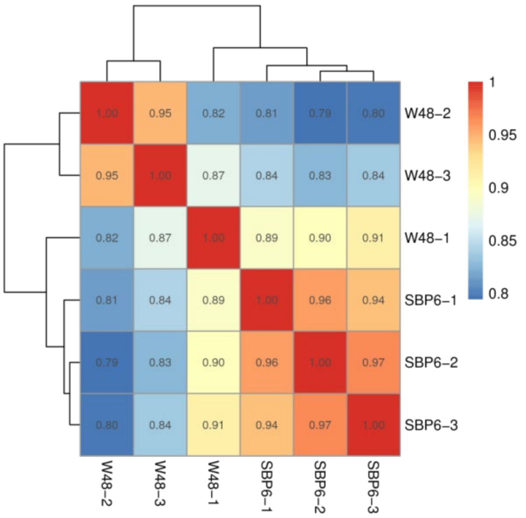

Figure S2. Transcription level correlation analysis

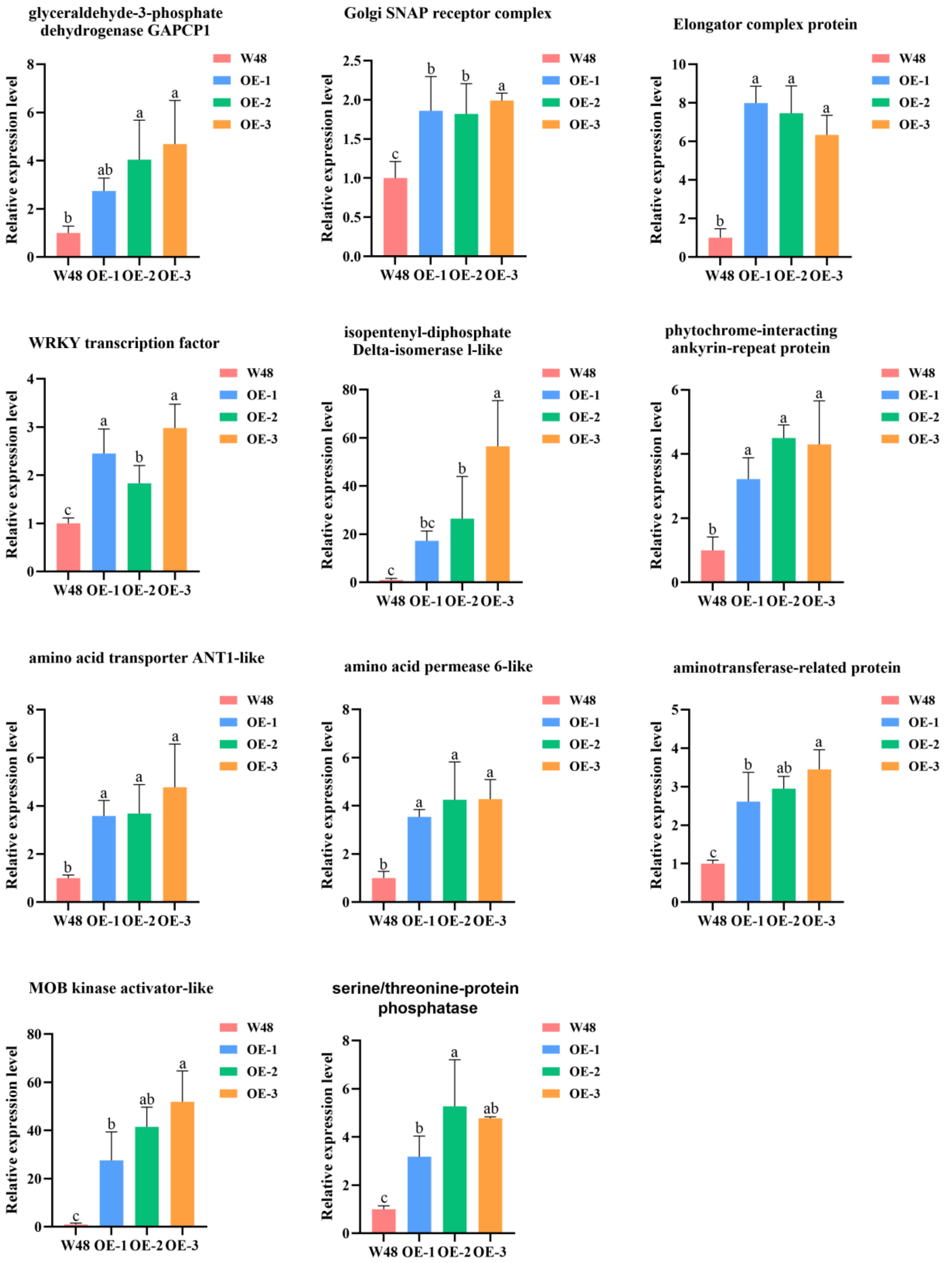

Figure S3. Transcription level verification of some differentially expressed genes. The data are presented as the mean ± standard deviation (SD) of three biological replicates. Values with different letters differ significantly ($p < 0.05$).
